# Urinary iodine concentration and thyroid volume of pregnant women attending antenatal care in two selected hospitals in Ashanti Region, Ghana: a comparative cross-sectional study

**DOI:** 10.1186/s12884-018-1820-3

**Published:** 2018-05-15

**Authors:** Daniel Gyamfi, Yaw Amo Wiafe, Kwabena Owusu Danquah, Ernest Adankwah, Gertrude Akua Amissah, Angela Odame

**Affiliations:** 1 Department of Medical Laboratory Technology, Faculty of Allied Health Sciences, Kwame Nkrumah University of Science and Technology, Kumasi, Ghana; 2 Department of Sonography, Faculty of Allied Health Sciences, Kwame Nkrumah University of Science and Technology, Kumasi, Ghana

**Keywords:** Iodine deficiency, Excessive iodine intake, Pregnant women, Thyroid volume, Goitre

## Abstract

**Background:**

Iodine deficiency is a major public health problem affecting people worldwide, particularly pregnant women. Iodine requirements increase substantially during pregnancy making pregnant women vulnerable to iodine deficiency and its disorders such as abortions, stillbirths and pregnancy goitre as well as congenital abnormalities, cretinism and mental retardation in their children. The primary aim of this study was to evaluate the prevalence of iodine deficiency and goitre among pregnant women attending antenatal sessions at two selected hospitals in Ashanti region, Ghana.

**Methods:**

A cross-sectional study was carried out in 239 pregnant women who attended the antenatal clinic at Kwame Nkrumah University of Science and Technology (KNUST) Hospital or Ejura District Hospital, both in Ashanti Region, Ghana. Socio-demographic data and information related to iodine were captured using a questionnaire. Urinary iodine concentration (UIC) was determined on spot urine samples using the Sandell-Kolthoff reaction with ammonium persulfate as digesting agent. Each woman’s thyroid volume was also measured by ultrasonography.

**Results:**

The overall median UIC was 155.9 μg/L, indicating adequate iodine intake in the study population. However, goitre prevalence in the pregnant women was 11.3%, denoting mild iodine deficiency. The median UIC for pregnant women who attended KNUST Hospital was higher (163.8 μg/L) than that of Ejura District Hospital (149.0 μg/L). The proportion of women who did not consume iodised salt was significantly higher (*p* < 0.001) in Ejura District Hospital (71.2%) than KNUST Hospital (28.0%). In total, 47.3% of the pregnant women studied had a UIC < 150 μg/L. Only 16.3% knew about the increase in iodine requirement during pregnancy and 21.3% of them had knowledge of the effects of iodine deficiency during pregnancy with most (81.8%) knowing of pregnancy goitre.

**Conclusion:**

There is generally adequate iodine intake among the pregnant women, however, iodine deficiency and goitre still exist among some pregnant women. Thus, assessment and continuous monitoring of iodine nutritional status in pregnant women in the country are warranted. Also, intensification of effective public health campaigns (through radio and television) with regard to iodine utilization and its benefits in pregnancy are still recommended among Ghanaian pregnant women.

**Electronic supplementary material:**

The online version of this article (10.1186/s12884-018-1820-3) contains supplementary material, which is available to authorized users.

## Background

Iodine is an important component of the thyroid hormones which regulate the body’s metabolic rate and are essential for brain development [1], especially during the gestational period and early childhood [2]. Iodine deficiency during pregnancy results in several problems such as abortions, stillbirths, congenital abnormalities, cretinism, goitre, mental retardation, squinting and mutism [3, 4].

Pregnant women are vulnerable to iodine deficiency since iodine requirements increase substantially during pregnancy [5]. This increase is due to many factors such as an increased demand for thyroxine (T4) in the mother so as to sustain normal metabolism, transport of T4 as well as iodide from the mother to the foetus [2], and an enhanced renal clearance of iodide [6], thus putting pregnant women and their unborn children at high risk of developing a series of functional and developmental abnormalities commonly known as iodine deficiency disorders (IDD) [7]. Based on this increase, the iodine requirement recommended by World Health Organization (WHO) for pregnant women (250 μg/day) is higher than that for adults (150 μg/day) [8].

A prolonged low intake of iodine may cause the thyroid gland of pregnant women to attempt to be more active in order to maintain required levels of thyroid hormones in the blood [9]. This makes the thyroid gland increases in size in order to trap iodine, a condition commonly referred to as pregnancy goitre [10]. The effects of in utero iodine deficiency on the developing foetus may have life-long effects on the offspring [11]. Buxton and Baguune [12] reported that due to iodine deficiency, 120,000 children born each year in Ghana stand a risk of developing intellectual impairment. They also realised that nearly 15,600 (13%) of these children are severely impaired and unable to develop normally, resulting in annual productivity loss of about 22 million dollars [12].

The Declaration for the Survival, Protection and Development of Children, which occurred after the World Summit for Children organized by the United Nations in 1989, stated that every mother is entitled to sufficient iodine nutrition to guarantee the normal brain development of her new-born child [13]. Fortification of salt with iodine - Universal Salt Iodization (USI) - is the most widespread, long-term and effective preventive strategy against IDDs introduced by WHO since 1920 [14]. The USI programme was launched in Ghana in 1995 to improve the consumption of iodised salt [4]. Salt iodization programmes are able in most cases to supply adequate iodine to meet the needs of adults but may not provide enough to meet the needs of pregnant women [15]. It is, therefore, imperative to do a regular check on the pregnant women’s iodine status in a population [15]. In order to do this, the WHO and the International Council for the Control of Iodine Deficiency Disorders (ICCIDD) recommended the determination of urinary iodine concentration as a suitable marker for monitoring iodine status in a population as well as measurement of thyroid size by ultrasonography [16]. Urinary iodine concentration is a marker of very recent iodine intake, whereas thyroid volume reflects long-term status rather than current intake [16].

Therefore, it is against this background that this study was organized to evaluate the iodine status in pregnant women who attended antenatal care at Kwame Nkrumah University of Science and Technology (KNUST) Hospital or Ejura District Hospital using urinary iodine concentration and thyroid volume, and to assess the possible sources of iodine consumption, sources of information on iodine utilisation, and knowledge of the increased iodine requirement or the effects of iodine insufficiency during pregnancy.

## Methods

### Design

This is a cross sectional study to assess urinary iodine concentration and thyroid volume in pregnant women in various trimesters who attended antenatal clinic at KNUST Hospital or Ejura District Hospital from January to April 2016. Prior to the study, a sample size of 222 was determined based on a previous prevalence of iodine deficiency of 42.5% found in pregnant women in the central part of Ghana [17]. The estimate generated was based on a two-sided alpha level of 5%, a marginal error of 6.5% and a confidence interval of 95%. Ethical clearance was sought from and approved by the Committee on Human Research, Publications and Ethics (CHRPE), KNUST, School of Medical Sciences and Komfo Anokye Teaching Hospital. The ethical approval reference number is CHRPE/RC/058/16. Permission to undertake the study at the hospitals was also sought from the hospital management. Written informed consent was obtained from all participants recruited into the study.

### Setting

The study was carried out in two randomly selected hospitals in Ashanti Region, Ghana. These hospitals are KNUST and Ejura District Hospitals. The KNUST Hospital is located in Kumasi – the regional capital and the second largest city in Ghana. The KNUST Hospital is the medical arm of the university. The hospital caters for a population of over 200,000 including students, staff and dependants and about 150,000 people from over 30 surrounding communities, including Ayigya, Bomso, Ayeduase, Kotei, Boadi, Deduako, Oforikrom and Anloga. There is a turnover of about 75–100 antenatal cases each week.

The Ejura District Hospital is situated in Ejura, the capital of Ejura-Sekyedumase Municipal Assembly in the Ashanti Region. Ejura is about 96 km from the regional capital, Kumasi. Ejura District Hospital caters for a settlement population of about 39,121 including antenatal cases from surrounding towns such as Miminaso, Anyinasu, Babaso and Sekyedumase.

### Participants

Recruitment of participants was done on each clinic day. On each day, every potential participant was approached and those who consented were recruited. Participants were recruited consecutively without specific preference to the trimester of pregnancy. Pregnant women with a history of thyroid disease, on thyroid medication and those who have had thyroidectomy in addition to pregnant women who had a history of smoking [18] were to be excluded, but none of the women approached warranted exclusion. In total, two hundred and thirty–nine (239) women in different trimesters of pregnancy were recruited for the study; 93 from KNUST Hospital and 146 from Ejura District Hospital.

### Administration of questionnaire

A questionnaire were designed to obtain information on socio-demographics, medical history, the use of tobacco, sources of iodine utilisation and knowledge of iodine utilisation, knowledge of effects of iodine deficiency or increased iodine requirement during pregnancy (see Additional file 1). The questionnaire was pilot-tested at KNUST Hospital, one of the study sites, by 50 participants and questions were modified based on feedback received. The study questionnaire was administered to participants from both hospitals via face to face interview conducted by trained research staff. The first section of the questionnaire asked about socio-demographic data, in which the women provided descriptive background information, while the second section of the questionnaire was related to consumption of iodised salt at home, intake of marine fish and iodine supplements, and sources of information on iodine utilisation. The third section asked about knowledge of the increase in iodine requirement and the effects of iodine deficiency during pregnancy, as well as medical history and the use of tobacco.

### Urinary iodine determination

Spot morning urine samples were collected from the women into clean, sterile, leak-proof plastic urine sample containers. Samples were kept in an ice chest in the antenatal clinics and transported on the same day to the Micronutrient Research Laboratory at Cancer and Infection Research Facility, KNUST. The samples were stored at − 20 °C until analysis. The Micronutrient Research Laboratory is an active participant of Ensuring the Quality of Urinary Iodine Procedures (EQUIP) of the US Centres for Disease Control and Prevention implemented to ensure good laboratory practice and the production of reliable urinary iodine results worldwide [19]. Urinary iodine concentration (UIC) (μg/L) was determined spectrophotometrically using the Sandell-Kolthoff reaction with ammonium persulfate as the digesting agent, one of the three most common methods used by participants of EQUIP programme [20]. This method is based on the principle that iodide released after digestion of urine with ammonium persulfate catalyses the reduction of ceric ammonium sulphate (yellow) to its cerous form (colourless) in the presence of arsenious acid which is detected by the rate of disappearance of the colour [16]. Following the protocol described by EQUIP [20], the participants’ urine samples were run in addition to iodine standards (0, 20, 40, 80, 120, 200, and 300 μg/L of iodine prepared from potassium iodate), internal quality control (IQC) samples (low and high concentrations) obtained from pooled urine samples, and external quality control samples from EQUIP.

Briefly, 1 mL of ammonium persulfate was added to 250 μL of urine samples, standards and quality control samples in separate test tubes, vortexed and heated in JB Aqua 18 plus water bath (Grant, UK) at 91-95 °C for 1 h to digest the sample. After cooling to room temperature, arsenious acid (3.5 mL) was added to each tube and incubated for 15 min at room temperature. Ceric ammonium sulphate (400 μL) was then added, vortexed and incubated at room temperature. At exactly 30 min after the addition of the ceric ammonium sulphate, the absorbance was read using LI-722 Visible Spectrophotometer (Lasany, India) at 420 nm. Using the developed iodine standard curve, the urinary iodine concentrations in the samples were obtained and WHO/ICCIDD reference medians for urinary iodine concentration were used to classify iodine intake as deficient (< 150 μg/L), adequate (150–249 μg/L), more than adequate (250–499 μg/L) or excess (≥500 μg/L) [21]. The coefficients of variation of inter-assay of the IQC samples were 8.0 and 9.2% at concentrations of 120 μg/L and 256 μg/L, respectively.

### Assessment of thyroid volume

In both centres, thyroid ultrasound was performed for all participants by the same sonographer who had 4 years scanning experience using the same portable machine. All scans were performed with a portable Siemens-Acuson P300 (Siemens Company, USA) equipped with a 7.5–12 MHz linear array transducer. Briefly, the pregnant women were asked to lie in the supine position with hyperextension of the neck. An ultrasound scan of the thyroid gland was then performed. Each lobe of the thyroid gland was assessed separately by measuring the three dimensions (height x width x length). The total volume calculated is the sum of the volumes of the right and left lobes without the inclusion of the isthmus. A normal thyroid gland was classified as the detection of a homogenous thyroid gland which was less than or equal to the 18 mL upper limit for women [22]. A goitre was categorized as a diffuse enlargement of the thyroid gland of more than 18 mL [22]. Texture of the thyroid gland was classified as normal, nodular or diffuse [23].

### Data analysis

Data were entered into Microsoft Excel, version 2010 (Microsoft Corporation, USA) and statistical analysis was performed using SPSS Statistics for Windows, version 21 (Armonk, NY: IBM Corp). Simple descriptive statistics such as frequencies and percentages and inferences based on chi-square test were performed on categorical variables such as age categories, stage of trimester, level of education, iodine use, and categories of iodine concentration. Ages of pregnant women were put into 6 categories with a class interval of 5. Kolmogorov-Smirnov test was used to determine the normality of the distribution of numeric data values. Consequently, non-parametric test (Mann-Whitney U test) was utilized to find the association between urinary iodine concentration and categories of thyroid volumes (≤18 mL and > 18 mL). Also, the correlation between two continuous variables (urinary iodine concentration and thyroid volume) was performed using Spearman rank correlation. The iodine status of the study population was calculated as median (interquartile range [IQR]) for UIC and thyroid volume. A two-tailed *p* value of less than 0.05 was considered as statistically significant.

## Results

### Socio-demographic and pregnancy-related characteristics of the participants

Of the total number of participants (239), 146 of the pregnant women attended antenatal clinic at Ejura District Hospital and 93 pregnant women at the KNUST Hospital (Table 1). The mean age of the study population was 27.6 ± 6.19 years. Out of the 6 age-categories, most of the women were in the 20–25 (33.5%) and 26–30 (35.6%) age ranges. Majority of the pregnant women were in their third trimester (46.9%) with most of them having no level of formal education (31.8%). The proportion of women with no level of formal education was higher (*p* < 0.001) at Ejura District Hospital (45.9%) than KNUST Hospital (9.7%).

**Table 1:** Socio-demographic and pregnancy-related characteristics of the pregnant women

	KNUST Hospital	EJURA Hospital	Total	*P* value^*^
	*n* (%)	*n* (%)	*n* (%)	
Age (Years)				0.072
< 20	4 (4.3)	13 (8.9)	17 (7.1)	
20–25	25 (26.9)	55 (37.7)	80 (33.5)	
26–30	33 (35.5)	52 (35.6)	85 (35.6)	
31–35	17 (18.3)	15 (10.3)	32 (13.4)	
36–40	12 (12.9)	6 (4.1)	18 (7.5)	
≥ 40	2 (2.1)	5 (3.4)	7 (2.9)	
Total	93 (100)	146 (100)	239 (100)	
Mean age (years)	28.9	26.7	27.6	0.063
Trimester				0.025
First	29 (31.2)	24 (16.4)	53 (22.2)	
Second	27 (29.0)	47 (32.2)	74 (31.0)	
Third	37 (39.8)	75 (51.4)	112 (46.9)	
Total	93 (100)	146 (100)	239 (100)	
Level of Education				< 0.001
None	9 (9.7)	67 (45.9)	76 (31.8)	
Primary	2 (2.2)	29 (19.7)	31 (13.0)	
JHS	28 (30.1)	32 (21.9)	60 (25.1)	
SHS	35 (37.6)	10 (6.9)	45 (18.8)	
Tertiary	19 (20.4)	8 (5.5)	27 (11.3)	
Total	93 (100)	146 (100)	239 (100)	

JHS: Junior High School; SHS: Senior High School

^*^p values are based on chi-square test

### Urinary iodine concentration and thyroid volume in the study population

The overall median urinary iodine concentration (UIC) of the pregnant women was 155.9 μg/L (109.4–235.3), showing an adequate iodine intake. However, the median UIC was higher in pregnant women who attended the antenatal clinic at KNUST Hospital [163.8 μg/L (107.3–258.4)] [adequate iodine intake] than those who attended the Ejura District Hospital [149.0 μg/L (108.9–224.4)], though the difference was not statistically significant. In total, 47.3% of the pregnant women studied had a UIC < 150 μg/L. Only 30.5% of the pregnant women had a UIC of 150–249 μg/L with 21.3 and 0.8% recording UIC of 250–499 μg/L and ≥ 500 μg/L, respectively. The proportion of pregnant women with UIC < 150 μg/L was higher in Ejura District Hospital (50.7%) than KNUST Hospital (41.9%), however, the difference was not statistically significant (Table 2). Also, the proportion of women who did not use iodised salt was significantly higher (*p* < 0.001) in Ejura District Hospital (71.2%) than KNUST Hospital (28.0%).

**Table 2:** Comparison of iodine status and sources of iodine utilisation of pregnant women who attended KNUST Hospital and Ejura District Hospital

	KNUST Hospital	EJURA Hospital	Total	*P* value^*^
	*n* (%)	*n* (%)	*n* (%)	
Urinary Iodine Concentration (μg/L) (*n* = 239)				0.221
< 150	39 (41.9)	74 (50.7)	113 (47.3)	
150–249	29 (31.2)	44 (30.1)	73 (30.5)	
250–499	25 (26.9)	26 (17.8)	51 (21.3)	
≥ 500	0 (0.0)	2 (1.4)	2 (0.8)	
Use of Iodised Salt (n = 239)				< 0.001
Yes	67 (72.0)	42 (28.8)	109 (45.6)	
No	26 (28.0)	104 (71.2)	130 (54.4)	
Marine Fish Consumption (n = 239)				0.295
Yes	91 (97.8)	139 (95.2)	230 (96.2)	
No	2 (2.2)	7 (4.8)	9 (3.8)	
Usage of iodine supplement (n = 239)				0.777
Yes	2 (2.2)	4 (2.7)	6 (2.5)	
No	91 (97.8)	142 (97.3)	233 (97.5)	

^*^p values are based on chi-square test

The median thyroid volume of the study population was 8.4 mL (6.5–10.9). Prevalence of goitre among the pregnant women was 11.3%. About 2.9% had diffuse goitre whilst 8.4% had nodular goitre. In the goitrous population, the prevalence of diffuse goitre was higher among pregnant women who attended Ejura District Hospital (38.5%) as compared to pregnant women who attended KNUST Hospital (14.3%). There was slightly larger proportion of women with thyroid volume > 18 mL in the second and third trimesters (4.1 and 2.7%, respectively) than the first trimester (1.9%). A greater number of women (4.6%) who did not use iodised salt had thyroid volume of > 18 mL, compared to those (0.9%) who used iodised salt (Table 3). However, all these differences were not statistically significant.

**Table 3:** Thyroid volume and its relationship with trimester and use of iodised salt

	Thyroid volume ≤ 18 mL	Thyroid volume > 18 mL
	*n* (%)	*n* (%)
Trimester		
First (*n* = 53)	52 (98.1)	1(1.9)
Second (*n* = 74)	71 (96.0)	3 (4.1)
Third (*n* = 112)	109 (97.3)	3 (2.7)
Use of Iodised Salt		
Yes (*n* = 109)	108 (99.1)	1 (0.9)
No (*n* = 130)	124 (95.4)	6 (4.6)

### Relationship between urinary iodine concentration and other parameters studied

The study observed a clinically significant negative weak correlation between urinary iodine concentration and thyroid volume (*r* = − 0.142; *p* = 0.028). A greater proportion of pregnant women with lower UIC (< 150 μg/L) had thyroid volume > 18 mL (71.4%) whereas 46.6% of pregnant women with lower UIC had thyroid volume ≤ 18 mL (Table 4). Pregnant women who had a UIC of 150–249 μg/L with thyroid volume ≤ 18 mL were 31.0% as against 14.3% pregnant women who had thyroid volume > 18 mL. The median UIC was 132.5 μg/L (44.6–221.0) and 156.9 μg/L (110.8–236.8) in pregnant women who had thyroid volume > 18 mL and ≤ 18 mL, respectively. However, the difference was not statistically significant.

**Table 4:** Relationship of urinary iodine concentration with various parameters

	Urinary Iodine Concentration [*n* (%)]					*P* value
	< 150(μg/L)	150–249(μg/L)	250–499(μg/L)	≥500(μg/L)	TOTAL	
Age group (years)						0.801
< 20	7 (41.2%)	9 (52.9%)	1 (5.9%)	0 (0.0%)	17 (100%)	
20–25	44 (55.0%)	20 (25.0%)	15 (18.8%)	1 (1.3%)	80 (100%)	
26–30	36 (42.4%)	30 (35.3%)	19 (22.4%)	0 (0.0%)	85 (100%)	
31–35	15 (46.9%)	9 (28.1%)	8 (25.0%)	0 (0.0%)	32 (100%)	
36–40	9 (45.0%)	3 (15.0%)	8 (40.0%)	0 (0.0%)	20 (100%)	
> 40	2 (40.0%)	2 (40.0%)	0 (0.0%)	1 (20.0%)	5 (100%)	
Trimester						0.755
First	23 (43.4%)	22 (41.5%)	8 (15.1%)	0 (0.0%)	53 (100%)	
Second	33 (44.6%)	19 (25.7%)	22 (29.7%)	0 (0.0%)	74 (100%)	
Third	57 (50.9%)	32 (28.6%)	21 (18.8%)	2 (1.8%)	112 (100%)	
Education						0.445
None	41 (54.0%)	21 (27.6%)	13 (17.1%)	1 (1.3%)	76 (100%)	
Primary	17 (54.8%)	8 (25.8%)	5 (16.1%)	1 (3.2%)	31 (100%)	
JHS	24 (40.0%)	25 (41.7%)	11 (18.3%)	0 (0.0%)	60 (100%)	
SHS	19 (42.2)	13 (28.9%)	13 (28.9%)	0 (0.0%)	45 (100%)	
Tertiary	12 (44.4%)	6 (22.2%)	9 (33.3%)	0 (0.0%)	27 (100%)	
Gravidity						0.562
< 3	62 (48.1%)	37 (28.7%)	29 (22.5%)	1 (0.8%)	129 (100%)	
3–5	43 (47.8%)	26 (28.9%)	20 (22.2%)	1 (1.1%)	90 (100%)	
> 5	8 (40.0%)	10 (50.0%)	2 (10.0%)	0 (0.0%)	20 (100%)	
Use Of Iodised Salt						0.115
Yes	46 (42.2%)	34 (31.2%)	28 (25.7%)	1 (0.9%)	109 (100%)	
No	67 (51.5%)	39 (30.0%)	23 (17.7%)	1 (0.8%)	130 (100%)	
Thyroid volume (ml)						0.028
≤ 18	108 (46.6%)	72 (31.0%)	50 (21.6%)	2 (0.9%)	232 (100%)	
> 18	5 (71.4%)	1 (14.3%)	1 (14.3%)	0 (0.0%)	7 (100%)	

*P* values are based on chi-square test

The age of the pregnant women, gestational age, level of education, gravidity and the use of iodised salt did not associate significantly with UIC (Table 4). However, the number of pregnant women with a UIC of 150–249 μg/L was higher in the first trimester (22/52) compared with the second (19/74) and third (32/112) trimesters. In addition, pregnant women who had no level of education recorded the highest number of women with a UIC < 150 μg/L (41/113). Similarly, a high number of pregnant women who did not use iodised salt had a UIC < 150 μg/L (67/113).

### Knowledge of increased iodine requirement or effects of iodine insufficiency and sources of information on iodine intake during pregnancy

Most of the participants (83.7%) had no knowledge of the increased iodine requirements during pregnancy (Table 5). Only 21.3% pregnant women out of the total study population had knowledge of the benefits of iodine consumption during pregnancy. It was observed that most of the women had no knowledge (86.2%) of the effects of iodine insufficiency in the mother or child. Out of those who had knowledge (13.8%), a greater proportion knew about pregnancy goitre (81.8%), followed by mental retardation (48.5%), cretinism (21.2%) and squinting (3.0%), but none had knowledge of both mutism and stillbirth (data not shown). Radio and television constituted the major sources of information on iodine and its benefits among the participants (91.6%). Only a few of the women (6.0%) had received information on iodine through health workers in their various hospitals and no pregnant woman had learnt about iodine from fliers or posters (Table 5).

**Table 5:** Knowledge of iodine requirement and iodine insufficiency effects and sources of information on iodine utilisation

	KNUST Hospital	EJURA Hospital	Total
	*n* (%)	*n* (%)	*n* (%)
Knowledge of increased iodine requirement			
Yes	17 (18.3)	22 (15.1)	39 (16.3)
No	76(81.7)	124 (84.9)	200(83.7)
Knowledge of effects of iodine insufficiency during pregnancy			
No	77 (82.8)	129 (88.4)	206 (86.2)
Yes	16 (17.2)	17 (11.6)	33 (13.8)
Sources of information			
Radio and television	76(81.7)	143(98.0)	219(91.6)
School	12(12.9)	2(1.4)	14(5.9)
Health Workers	5(5.4)	1(0.7)	6(2.5)
Fliers	0(0.00)	0(0.0)	0(0.0)
Posters	0(0.00)	0(0.0)	0(0.0)

## Discussion

Iodine is required for the synthesis of thyroid hormones, which are crucial regulators of early brain development. Iodine requirement increases during pregnancy and insufficiency during pregnancy can cause maternal hypothyroidism leading to pregnancy goitre and foetal hypothyroidism, which causes impaired neurological development of the foetus [1]. The study mainly sought to determine the urinary iodine concentration and thyroid volume in pregnant women who attended antenatal clinics at two selected hospitals - KNUST Hospital and Ejura District Hospital, all in Ashanti Region, Ghana.

Generally, the median UIC obtained in the study population (155.9 μg/L) indicates adequate iodine intake according to WHO criteria [20]. However, of the 239 pregnant women recruited, 113 (47.3%) had a UIC < 150 μg/L as judged by urinary iodine concentration. A similar finding was obtained in a recent study carried out among pregnant women by Simpong et al. [17] at Kissi Health Centre in the Central Region of Ghana where 42.5% of pregnant women were iodine deficient by the same method. However, Simpong et al. [17] did not assess the thyroid volume in their study. Our study showed goitre prevalence of 11.3%, indicating mild iodine deficiency among the pregnant women [24]. These results suggest that some Ghanaian pregnant women are still not meeting the higher iodine requirements during pregnancy; this may lead to a negative effect on foetal brain development [25–27] and thus a general survey of iodine nutritional status in pregnancy is recommended.

In this study, it was found that the iodine status of pregnant women who attended antenatal care at Ejura District Hospital (located far from the regional capital) was just below the cut-off for sufficiency as recommended by WHO, with median UIC of 149 μg/L (generally adequate) whereas pregnant women who attended antenatal care at the KNUST Hospital (found in the regional capital) had median UIC of 164 μg/L (adequacy). Additionally, thyroid volume was found to be increased in 38.5%(7) of pregnant women who attended antenatal at Ejura District Hospital as compared to 14.3% of pregnant women attending KNUST Hospital. The difference in the median UIC and thyroid volume in these areas can be attributed to iodised salt intake since consumption of table salt is the main factor that influences iodine intake in countries which have implemented the universal salt iodization programme [28]. This study found that iodised salt intake in pregnant women at Ejura District Hospital was 28.8% as compared to 72.0% seen in pregnant women at KNUST Hospital. A similar trend was reported by Habimana et al. [29] who also obtained median UIC to be lower in a suburban area than urban area in Republic of Congo. Sultanalieva et al. [30] and Simpong et al. [17] also concluded that the iodine nutritional status was highly responsive to the use of iodised salt. This means that there should be continuous monitoring and education among pregnant women on the need to use iodised salt.

It was observed in this study that the association between urinary iodine concentration and thyroid volume was clinically significant (*p* = 0.028). Median UIC was 138.2 μg/L in pregnant women who had thyroid volume > 18 mL and 155.9 μg/L in those who had ≤18 mL. Research conducted in various countries to assess maternal urinary iodine concentration and thyroid volume indicated that in iodine deficient pregnant women, thyroid volume increased significantly [31–35]. During pregnancy, the thyroid becomes more active leading to the production of about 50% more thyroid hormones than usual [36]. Giusti et al. [10] stated that a reduced intake of iodine for a long period of time may stimulate the thyroid gland of pregnant women to be more active to maintain the correct level of thyroid hormones in the blood. This means that the thyroid increases in size in order to trap more iodine, which leads to pregnancy goitre (thyroid volume > 18 mL). This could account for the increased thyroid volume seen in pregnant women with UIC < 150 μg/L in this study.

The finding of slightly higher thyroid volume values during the second and third trimesters of pregnancy than the first trimester is consistent with previous reports from other researchers [29, 31, 37]. This could be explained by the increase in oestrogen concentration during pregnancy which peaks during the third trimester to enable the uterus and placenta to improve vascularization [38]. Oestrogen induces increased synthesis of thyroid hormone binding proteins and since greater than 99% of thyroid hormones in the blood are attached to these proteins, the total thyroid hormone concentration in the blood consequently increases [39]. This implies that more iodine is needed for the increased thyroid hormone synthesis across the first trimester to the third [5]. A relatively low iodine intake during pregnancy with time, leads to thyroidal stress, with increases in thyroid size [40]. This thus calls for education and monitoring of iodine status in pregnant women during early stage of the pregnancy and thereafter.

Only fifty-one (21.3%) of the pregnant women had knowledge of the effect of iodine deficiency during pregnancy with knowledge of pregnancy goitre being the highest (81.8%). A similar study performed at Kaneshie Polyclinic in the Greater Accra Region of Ghana revealed that 79.4% of pregnant women knew about pregnancy goitre more than the other effects [41]. Most participants reported getting knowledge of iodine intake from radio and television (91.6%). A few obtained the knowledge from school (5.9%) and health workers at their hospitals (2.5%). However, a study in Australian pregnant women showed that most pregnant women obtained knowledge of iodine and its effects from their healthcare professionals (49.0%) at various health centres, with 35.2% of them not having any knowledge of iodine [42]. This indicates that public health campaign strategic on the importance of iodine consumption involving radio and television needs to be utilised more in Ghana besides encouraging health workers in our antenatal care centres to intensify the education on iodine and its importance during pregnancy.

## Conclusion

These findings suggest that after more than two decades of the implementation of the universal salt iodisation programme in Ghana, iodine intake in our study population is adequate, however, iodine deficiency still exists in some pregnant women and these women are at risk of iodine deficiency disorders. Also, utilization of iodised salt as well as knowledge of increased requirement of iodine during pregnancy is generally low among the women especially those in areas far from the regional capital. Efforts need to be intensified through better educational programmes on the benefits and increased use of iodised salt during pregnancy in such areas. Further research is recommended to look at the possible barriers to the use of iodised salt especially in areas far from the regional capitals. Also, longitudinal study can be conducted to find the changes in iodine status over the course of pregnancy in the women in our setting.

## Additional file

Additional file 1: Questionnaire. (PDF 34 kb)

## Abbreviations

CHRPE: Committee on Human Research, Publications and Ethics
EQUIP: Ensuring the Quality of Urinary Iodine Procedures
ICCIDD: International Council for the Control of Iodine Deficiency Disorders
IDD: Iodine deficiency disorders
IQC: Internal quality control
IQR: Interquartile range
KNUST: Kwame Nkrumah University of Science and Technology
T4: Thyroxine
UIC: Urinary iodine concentration
WHO: World Health Organization
