# Supplementary material for: Urinary iodine concentration and thyroid volume of pregnant women attending antenatal care in two selected hospitals in Ashanti Region, Ghana: a comparative cross-sectional study
Source: BMC Pregnancy Childbirth. 2018 May 15;18:166. doi: 10.1186/s12884-018-1820-3 (PMC5952633; doi:10.1186/s12884-018-1820-3)
Supplement: Supplementary file 1 — Questionnaire. (PDF 34 kb) [file 12884_2018_1820_MOESM1_ESM.pdf]

QUESTIONNAIRE FOR ASSESSING IODINE STATUS AND THYROID VOLUME IN PREGNANT WOMEN ATTENDING ANTENATAL CARE AT KNUST HOSPITAL OR EJURA DISTRICT HOSPITAL

Date of interview ____/_____/_____ Identification code _____________

Please provide complete and sincere answers as possible. Tick the best option. All information will be treated in strict confidence.

I. Socio-demographics and pregnancy background

1) Age (years) [ ] < 20 [ ] 20-25 [ ] 26-30 [ ] 31-35 [ ] 36-40 [ ] > 40

2) Level of education [ ] none [ ] Primary [ ] J.H.S. [ ] S.H.S. [ ] Tertiary

3) Trimester [ ] 1st trimester [ ] 2nd trimester [ ] 3rd trimester

4) Number of pregnancies [ ] < 3 [ ] 3-5 [ ] > 5

5) Number of stillbirths/abortions [ ] < 3 [ ] 3-5 [ ] > 5

II. Knowledge of use of iodised salt, sources of information on iodine utilisation and iodine deficiency disorders

6) Have you heard about iodised salt? [ ] Yes [ ] No

7) If yes, how did you learn about iodine utilisation?

   [ ] Posters/billboards [ ] Flyers [ ] Radio/television

   [ ] Health worker [ ] School

8) Do you use iodised salt? [ ] Yes [ ] No

9) Do you take herrings and other ocean fish?

   [ ] Yes [ ] No

10) Do you take iodine supplements? [ ] Yes [ ] No

11) Have you heard about iodine deficiency disorders (IDDs)?

   [ ] Yes [ ] No

III. Knowledge of increased iodine requirement and effects of iodine deficiency during pregnancy, medical history and the use of tobacco

12) Do you know that iodine requirements increase during pregnancy?

[ ] Yes [ ] No

13) Do you know of the effects of iodine deficiency during pregnancy?

[ ] Yes [ ] No

14) If yes, which of these effects do you know of?

[ ] Mental retardation [ ] Cretinism [ ] Squinting

[ ] Mutism [ ] Pregnancy goitre [ ] Miscarriage/stillbirth

15) Do you/have you had any thyroid disease? [ ] Yes [ ] No

16) Have you undergone thyroidectomy? [ ] Yes [ ] No

17) Are you on any thyroid medication? [ ] Yes [ ] No

18) Do you smoke? [ ] Yes [ ] No
